# Pediatric orbital emphysema and pneumocephalus following a compressed air gun injury: a case report

**DOI:** 10.1186/s12887-025-05468-0

**Published:** 2025-03-03

**Authors:** Ahmed Bassiouny, Amgad El Nokrashy, Mustafa Al sharabi, Baraa Al-Ashker, Eslam A. Elsery, Aya M. Hashish

**Affiliations:** 1 Diagnostic Radiology, Faculty of Medicine, Mansoura University, 60 El Gomhouria St, P.O. Box 35516, Mansoura, Egypt; 2 Ophthalmology department, Faculty of Medicine, Mansoura University, Mansoura, Dakahlia Egypt; 3 Mansoura University Hospitals, Faculty of Medicine, Mansoura University, Mansoura, Egypt

**Keywords:** Pneumocephalus, Orbital emphysema, Air gun injury, Sub-conjunctival hemorrhage

## Abstract

**Background:**

Orbital emphysema and pneumocephalus rarely occur without associated skull fractures. Over the past decades, a few case reports have documented compressed air injuries as a rare cause of orbital emphysema and pneumocephalus in the absence of concomitant skull fractures leading to various injuries.

**Case presentation:**

We illustrate here a 12-year-old boy who presented with painless left eye swelling following an accidental compressed air blast injury. On examination, he exhibited mild bilateral visual acuity impairment. The left eyelids, periorbital region, and cheeks were swollen, with swelling extending to the jaw with palpable crepitations. Ocular motility of the left eye was restricted in all directions. Slit lamp examination revealed subconjunctival hemorrhage, air bubbles, and chemosis, while the cornea, anterior chamber and lens were normal. Pupillary reactions were brisk. Fundus examination and intraocular pressure were normal. The right eye and adnexa were completely normal. The patient had no neurological symptoms. NCCT scan revealed left frontal and temporal subcutaneous emphysema, bilateral orbital emphysema, and multiple gas foci in the left masticator, parotid and carotid spaces, as well as in both parapharyngeal spaces. Pneumocephalus was noted in the left temporal lobe, along the left cavernous sinus and parasellar region without bony fractures. The patient underwent surgical exploration of the sclera of the left globe under general anesthesia to exclude the presence of a scleral wound under subconjunctival hemorrhage and air bubbles and he was free. The patient was managed conservatively with instructions for bed rest, head elevation, and avoidance of the Valsalva maneuver. Gradual improvement was reported over follow-up, with the patient returning to normal after four weeks.

**Conclusions:**

Both orbital emphysema and pneumocephalus can occur in rare instances without skull fractures. The symptoms can range from mild to life-threatening. Mild cases can be managed conservatively.

## Background

Orbital emphysema and pneumocephalus often occur due to transmission of air into the orbit and intracranially following traumatic skull fractures [1, 2]. Compressed air injuries, typically caused by air guns, have been reported as a rare cause of orbital emphysema and pneumocephalus and are often related to industrial accidents in male adults, leading to various injuries [3–5]. Only a few pediatric cases were reported in literature [4–9]. Here we present a rare case of orbital emphysema and pneumocephalus following a compressed air injury without skull fractures.

## Case presentation

**Fig. 1:**
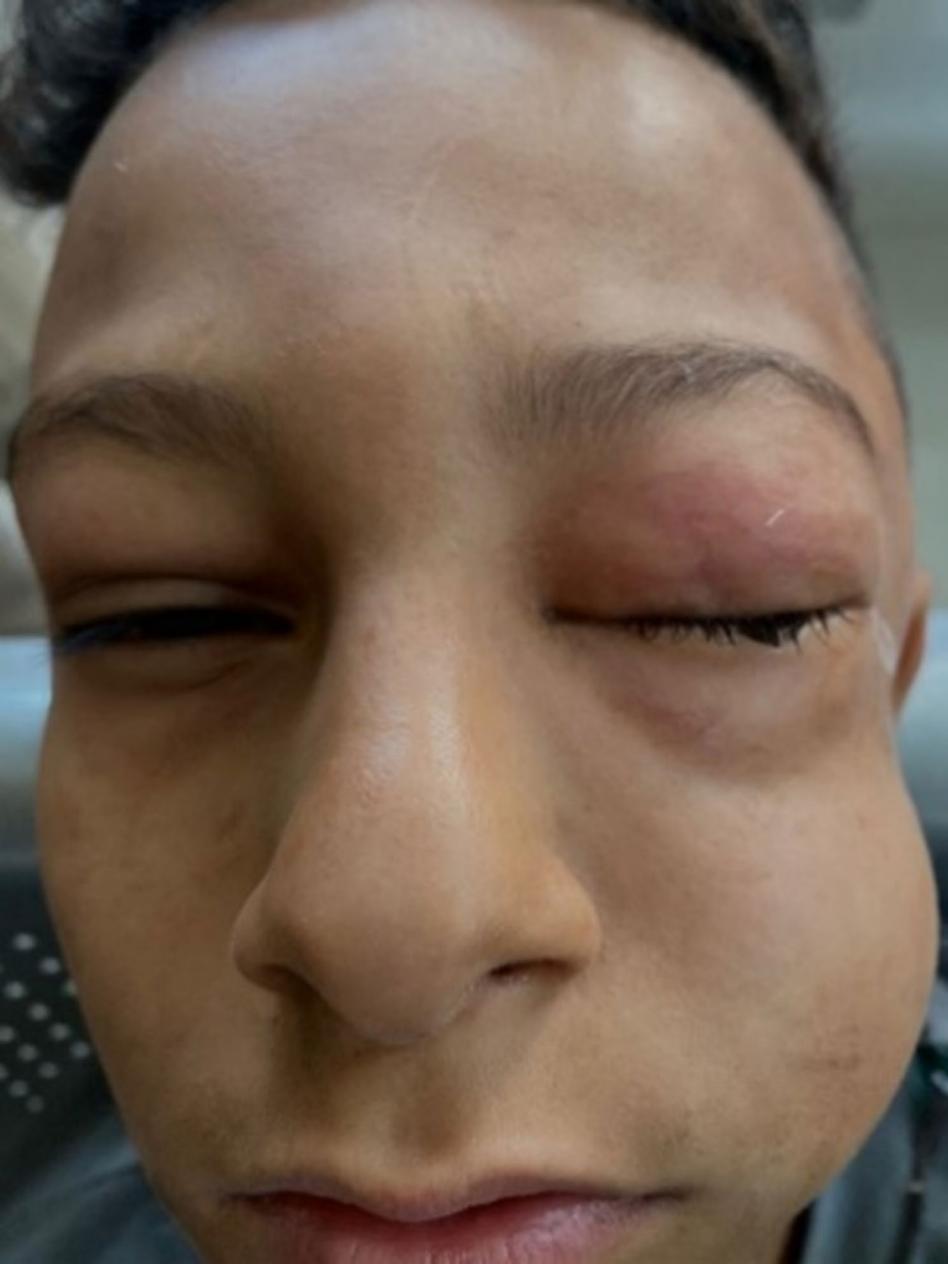
Clinical examination of the patient showing left eyelids, periorbital, cheek and jaw swelling. (Reproduced with written informed consent for publication obtained from the patient’s parents)

A 12-year-old boy without any past medical history presented to Mansoura Ophthalmic Center emergency department complaining of painless left eye swelling following an accidental compressed air blast injury. On clinical examination, his visual acuity was 6\18 in his left and 6/9 in the right eye. The left eyelids, periorbital region, and cheeks were swollen with swelling extending to the jaw (Fig. 1). On palpation of the periorbital region of left eye, crepitations were felt along the swelling. Ocular motility of the left eye was limited in all directions. Slit lamp examination revealed sub-conjunctival hemorrhage, air-bubbles, and chemosis. The remainder of the ocular examination was normal, with brisk direct and consensual pupillary reactions. Fundus examination and intraocular pressure (IOP) of the left eye were normal. The right eye and its adnexa were normal, except for diffuse periorbital swelling. The patient reported no neurological symptoms.

**Fig. 2:**
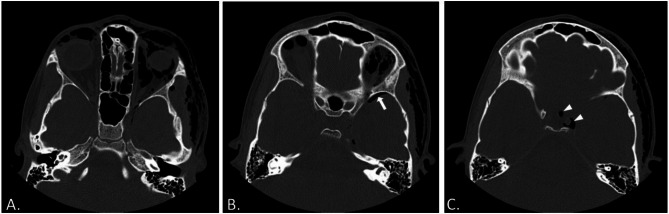
(**A**) Non-contrast axial CT of the brain and orbits demonstrating subcutaneous emphysema, bilateral orbital emphysema, with gas foci noted along the left superior orbital fissure. (**B**) Pneumocephalus observed in the left temporal lobe (arrow). (**C**) Cranial cuts showing gas foci along the left cavernous sinus and left parasellar area (arrow heads)

The patient underwent computerized tomography, which revealed left frontal and temporal subcutaneous emphysema, bilateral orbital and preseptal gas foci, along with left orbital emphysema. A streak of gas foci was noted along the superior orbital fissure. Multiple gas foci were present in the left masticator, parotid, and carotid spaces, as well as in both parapharyngeal spaces. Pneumocephalus was observed at the left temporal lobe, along the left cavernous sinus and in the parasellar region. No bony fractures were reported (Fig. 2).

The patient underwent surgical exploration of the sclera of the left globe under general anesthesia to exclude the presence of a scleral wound beneath the subconjunctival hemorrhage and air bubbles; no scleral wound was found. The patient was prescribed medical treatment in the form of topical combined antibiotic and steroid eye-drops, to be administered five times daily. He was instructed to have complete bed rest with head elevation at 30^o^ and to avoid the Valsalva maneuver.

On his first day follow-up, the visual acuity of the left and right eyes was 6\9 and 6\6, respectively. On the second follow-up after one week, slit lamp examination revealed minimal hyphema (1 mm) which was treated medically with topical combined antibiotic and steroid eye drops administered five times daily. One week later, the hyphema was resolved and treatment was gradually withdrawn and discontinued by the fourth follow-up after four weeks. There were no neurological symptoms at any of his follow-ups.

## Discussion and conclusions

Orbital emphysema commonly occurs as a result of medial orbital wall fractures [3]. In compressed air injuries, the high air pressure pushes air through the subcutaneous and the subconjunctival space into retrobulbar space. Pneumocephalus following orbital trauma in absence of skull fractures has been suggested by two mechanisms; It can enter the extradural space through the superior orbital fissure [10] or it can dissect the Tenon fascia surrounding the optic nerve and enter the subarachnoid space and ventricles through the optic canal [11]. We believe pneumocephalus occurring in our case occurred by the first mechanism, as evidenced by the presence of air near the superior orbital fissure and the sella. There were no skull fractures on CT, nor were there clinical signs suggestive of CSF leakage.

Despite the severity of emphysema in this case, no orbital bony fractures were detected. Orbital emphysema is usually a benign and self-limiting condition but can also result in more serious clinical outcomes, such as proptosis, loss of vision, increased intraocular pressure, and central retinal artery occlusion. Simple cases are typically treated conservatively, whereas more severe symptoms may require interventions such as IV antibiotics, steroids, needle or surgical decompression [12]. Our patient reported mild symptoms, and scleral wounds were excluded on surgical exploration. During the first follow-up on the first day after surgical scleral exploration, there was no evidence of hyphema. At the second follow-up, one-week post-surgery, a minimal hyphema was observed. The initial presence of hyphema cannot be definitively excluded, as it could have been masked by eyelid tension or may have initially dispersed. The patient was therefore managed conservatively.

Patients with simple pneumocephalus often present with a headache or non-specific complaints. However, those with a more life-threatening tension pneumocephalus can have sensory deficits, papilledema, respiratory irregularities, or cardiac arrest in case of mass effect or herniation [4, 13]. Conservative therapy is the initial treatment of simple pneumocephalus. Neurosurgical intervention is indicated in severe, recurrent, or, persistent cases [14]. Our patient reported no neurological symptoms and had minor foci of pneumocephalus; thus, he was managed conservatively.

In conclusion, orbital emphysema and pneumocephalus can occur without traumatic skull injuries. In our case, we demonstrated both occurring following a compressed air injury. Mild cases can be managed conservatively, with patients typically returning to normal within a few weeks. Continuous monitoring and follow-up are advised to mitigate potential complications.

## Data Availability

The data and images used in this case report are available from the corresponding author on reasonable request.
